# Cross-sectoral antimicrobial resistance patterns of *Escherichia coli* across human, animal, and environmental interfaces in southern Bangladesh

**DOI:** 10.14202/vetworld.2026.1970-1983

**Published:** 2026-05-12

**Authors:** Rahima Akther Dipa, Ibrahim Khalil, Md Sabbir Hossen, Md Nurul Alam, AKM Akbar Kabir, Md Mynul Hasan Jesan, Kamrun Nahar Koly, Pk Niharjon, Mohammad Mahmudul Hassan

**Affiliations:** 1 Section of Physiology, Department of Preclinical Sciences, Habiganj Agricultural University, Habiganj, Bangladesh; 2 Field Disease Investigation Laboratory, Barishal, Department of Livestock Services, Ministry of Fisheries and Livestock, Bangladesh; 3 Faculty of Animal Science and Veterinary Medicine, Patuakhali Science and Technology University, Patuakhali, Bangladesh; 4 Department of Microbiology, Sher-E-Bangla Medical College, Barishal, Bangladesh; 5 Department of Physiology, Biochemistry and Pharmacology, Faculty of Veterinary Medicine, Chattogram Veterinary and Animal Sciences University, Chattogram, Bangladesh

**Keywords:** antimicrobial resistance, azithromycin resistance, ciprofloxacin resistance, *Escherichia coli*, One Health approach, poultry farming, urinary tract infection, zoonotic transmission

## Abstract

**Background and Aim:**

Antimicrobial resistance (AMR) is an escalating global health concern, particularly in low- and middle-income countries where human, animal, and environmental interfaces are closely interconnected. This study aimed to compare AMR patterns in *Escherichia coli* isolates across human and animal health sectors within a shared ecological setting in southern Bangladesh, adopting a One Health perspective.

**Materials and Methods:**

A retrospective cross-sectional study was conducted in Barishal District, Bangladesh, using routine surveillance data collected between January and August 2023. A total of 217 samples were analyzed, including 125 human urine samples and 92 animal-associated samples (poultry liver and farm environmental sources). Isolation and identification of *E. coli* were performed using standard culture and biochemical methods. Antimicrobial susceptibility testing was conducted using the Kirby–Bauer disk diffusion method against ampicillin, ciprofloxacin, and azithromycin. Statistical analyses included univariate comparisons, multivariate logistic regression, and principal component analysis (PCA) to assess resistance patterns and sectoral differences.

**Results:**

*E. coli* was isolated in 54.4% of human samples and 53.3% of animal-associated samples. Human isolates exhibited significantly higher resistance to ampicillin (86.8% vs. 46.9%, p < 0.001) and azithromycin (55.9% vs. 8.2%, p < 0.001) compared to animal isolates, while ciprofloxacin resistance showed no significant difference (39.7% vs. 28.6%, p = 0.243). Multivariate analysis revealed significantly higher odds of resistance in human isolates for ampicillin (OR = 4.3) and azithromycin (OR = 9.3). PCA demonstrated that resistance to ampicillin and azithromycin was the primary driver of variability, collectively explaining over 80% of the observed variance. Human isolates clustered distinctly, indicating stronger resistance profiles, whereas animal isolates showed greater variability, particularly for ciprofloxacin.

**Conclusion:**

This study demonstrates clear sectoral differences in AMR patterns of *E. coli* in southern Bangladesh, with human isolates showing markedly higher resistance to key antibiotics. These findings underscore the urgent need for integrated One Health strategies, strengthened antimicrobial stewardship, and enhanced surveillance systems. Addressing AMR requires coordinated efforts across human, animal, and environmental health sectors to mitigate the spread and impact of resistant pathogens.

## INTRODUCTION

Alexander Fleming, the inventor of penicillin and a pioneer in the era of antimicrobials, once warned that the overuse of penicillin would lead to the emergence of resistant bacteria, rendering this life-saving medication ineffective [1]. As predicted, antimicrobial resistance (AMR) has become a global concern [2]. The rise of AMR poses significant threats to both public health and food security. AMR undermines the effectiveness of medical treatments, leading to more severe infections, increased mortality rates, and escalating healthcare costs [3]. In animals, particularly in poultry farming, the use of antibiotics for growth promotion and disease prevention contributes to the emergence of resistant bacteria [4], which can be transmitted to humans through contaminated food, thereby compromising food safety [5]. In Bangladesh, AMR has emerged as a critical public health challenge. Recent studies reported high rates of resistance in common pathogens. For urinary tract infections (UTIs), *Escherichia coli* exhibits resistance to ciprofloxacin (CIP) ranging from 43–66% and to ampicillin (AMP) up to 83% [6, 7]. A study of uropathogens from complicated UTIs found that *E. coli* (49%) and *Klebsiella pneumoniae* (25%) were the predominant isolates, with significant resistance to quinolones and cephalosporins [8]. In poultry production, antimicrobial use is widespread, with a recent study documenting that birds receive antimicrobial treatment for approximately 60% of their lifetime, with fluoroquinolones, sulfonamides, and aminopenicillins being the most frequently used classes [9]. This high level of antibiotic exposure in poultry contributes to the emergence and dissemination of resistant bacteria, posing risks to human health through foodborne transmission and environmental contamination [10, 11]. While recent genomic One Health studies have tracked antimicrobial resistance gene (ARG) transmission in Dhaka and rural settings, phenotypic cross-sectoral comparisons in southern Bangladesh, particularly integrating farm environmental reservoirs alongside clinical human and diagnostic animal samples, remain limited [12].

In response to these threats, Bangladesh has enacted various regulations to manage the use of antimicrobials in both human and veterinary medicine. The country’s National Action Plan for AMR incorporates a One Health approach, emphasizing the interconnection between human, animal, and environmental health [2]. In veterinary practice, the use of certain antibiotics, such as CIP and azithromycin (AZM), is restricted to prevent their misuse in livestock production, as described in [13]. Despite these regulatory efforts, the misuse of antibiotics remains widespread due to weak enforcement, limited public awareness, and inadequate surveillance, further exacerbating the AMR crisis. In southern Bangladesh, particularly in Barishal, high-density poultry farms exacerbate antibiotic misuse, contributing to the rise of AMR [14].

*E. coli*, a Gram-negative organism, exacerbates public health risks by acquiring and transferring resistant genes to other bacteria, thereby demonstrating resistance to multiple antibiotics in both humans and animals [15]. Studies have reported *E. coli* resistance in humans to AMP (82.8%), CIP (67.9%), and AZM (40.4%) [16, 17]. Additionally, a clinical study found high resistance to penicillin (98.2%) and AMP (97.3%) [18]. In poultry, *E. coli* exhibited resistance to AMP in layers (54%) [19], CIP in broilers (44.5%), and AZM in broilers (>10%) [20]. In farm environments, *E. coli* demonstrated significant resistance to CIP (88.8%) [21] and AMP (77.5%) [22]. These findings highlight the interconnected nature of AMR across human, animal, and environmental sectors.

Resistance patterns at the human–animal–environment interface underscore the necessity of an integrated understanding of AMR dynamics. While extensive research has independently explored AMR in human and animal populations, most studies remain sector-specific and do not provide direct comparative insights across these interconnected domains. Although recent genomic investigations have improved understanding of ARG transmission, phenotypic comparisons that simultaneously incorporate human clinical samples, animal diagnostic samples, and farm environmental reservoirs within a shared ecological setting remain scarce. This limitation is particularly evident in resource-limited regions such as southern Bangladesh, where integrated One Health surveillance systems are still evolving.

Furthermore, existing studies often differ in the selection of antibiotics tested across sectors, limiting the ability to identify consistent resistance drivers and sector-specific selective pressures. The lack of harmonized, cross-sectoral phenotypic data restricts evidence-based decision-making for antimicrobial stewardship and policy interventions. Therefore, there is a critical need for studies that directly compare AMR patterns across human, animal, and environmental interfaces within the same geographic and ecological context to better understand the dynamics of resistance dissemination.

The present study aims to compare AMR patterns in *E. coli* isolates from human and animal health settings in the Barishal District of Bangladesh, focusing on targeted antimicrobials such as AMP, CIP, and AZM. These antibiotics were selected due to their widespread use in both human and veterinary settings in Bangladesh and their prioritization for monitoring in the National Action Plan (NAP) on antimicrobial resistance. In addition, AMP belongs to the World Health Organization (WHO) AWaRe “Access” group, while CIP and AZM are categorized under the “Watch” group, highlighting their importance for surveillance and stewardship.

However, other critically important antibiotics, such as colistin, third- and fourth-generation cephalosporins, and carbapenems, which are also relevant in regional *E. coli* resistance, were not included in this study due to limitations in routine diagnostic testing and surveillance scope. The study hypothesized that resistance rates would be higher in human isolates compared to animal isolates, reflecting greater therapeutic antibiotic pressure in human healthcare settings. Specifically, it was anticipated that AMP and AZM resistance would show the most pronounced sector-wise differences due to their extensive use in human medicine. By testing this hypothesis and highlighting sector-wise differences in resistance patterns, this study supports the need for integrated One Health approaches to strengthen antibiotic stewardship and AMR surveillance systems.

## MATERIALS AND METHODS

### Ethical approval

This study was approved by the Institutional Review Board of Sher-E-Bangla Medical College, University of Dhaka, under memo number 59.14.0000.130.99.001.24.1590, dated 12 August 2024. Ethical clearance was obtained, and the dataset was anonymized to ensure participant confidentiality, with no personally identifiable information included. Given the retrospective nature of the data, informed consent was not required. The study adhered to the ethical principles of the Declaration of Helsinki and complied with relevant data protection regulations. No conflicts of interest related to ethics are declared. Poultry samples were collected post-mortem from birds submitted to the Field Disease Investigation Laboratory (FDIL) for routine diagnostic purposes. No live animals were used for experimental procedures in this study, and animal sampling followed institutional guidelines for diagnostic necropsy. This sampling was exempt from formal ethics review as it relied on routine diagnostic submissions.

### Study period and location

**Figure 1:**
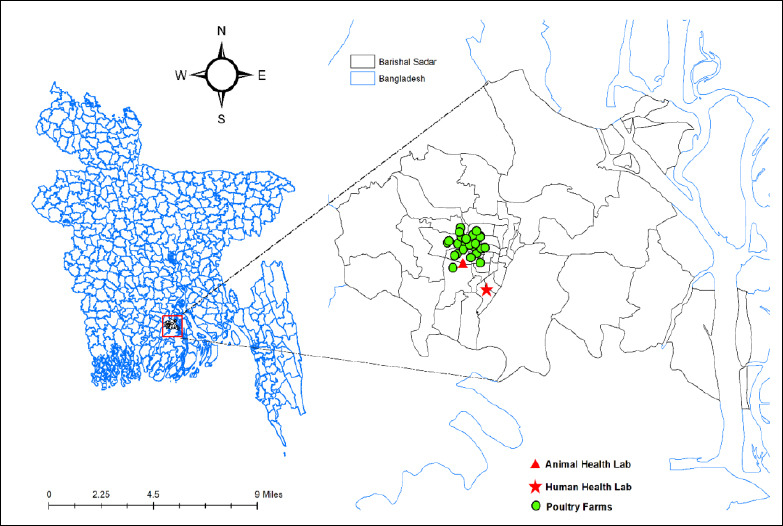
Locations of the Animal Health Laboratory (▲), Human Health Laboratory (★), and poultry farms (●) within the Barishal District, Bangladesh. The map highlights the proximity of these sites, all situated near one another, providing a shared ecological and geographic context for comparing AMR patterns across human, animal, and environmental health sectors.

The study was conducted in Barishal District, southern Bangladesh, between 01 January and 31 August 2023. The human and animal health laboratories, along with the poultry farms included in this study, were located within an approximately 10 km radius in Barishal District, allowing comparison of resistance patterns in a shared geographic and ecological context (Figure 1).

### Study design

A retrospective cross-sectional study was conducted to compare AMR patterns of *E. coli* isolates obtained from human and animal health settings within a One Health framework. Human urine samples and poultry submissions ensured an accurate representation of both sectors. This cross-sectional design allowed direct comparison of contemporaneous resistance patterns across human, animal, and environmental sectors using existing surveillance infrastructure. Data used in this study were derived from routine laboratory surveillance activities conducted independently in human and animal health sectors. This observational study was reported in accordance with the STROBE guidelines (Supplementary Table S1).

### Sample collection

In this study, samples were collected from three distinct sources: animal health, farm environment, and human health, to compare AMR patterns in *E. coli* isolates.

**Figure 2:**
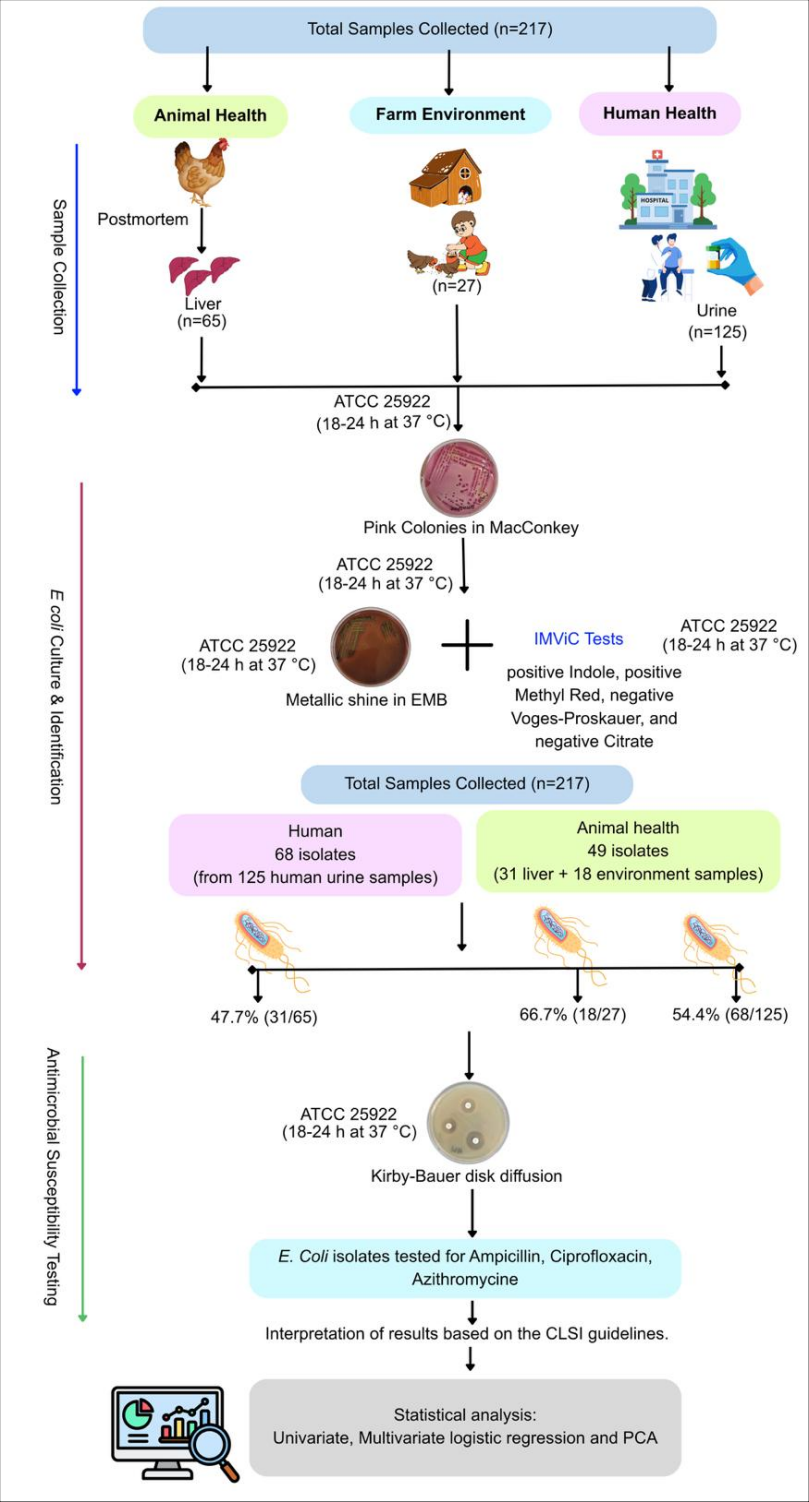
Schematic overview of sample collection, *Escherichia coli* identification, and antimicrobial susceptibility testing across human, animal, and farm environmental sources.

Animal health samples: A total of 65 poultry samples were received from birds submitted for diagnostic investigation at FDIL in Barishal, Bangladesh. Liver samples were collected post-mortem for disease diagnostic purposes. These liver samples were subsequently sent for microbial isolation and antimicrobial sensitivity testing to assist in determining effective treatment options (Figure 2).

Farm environmental samples: In addition to the animal health samples, 27 active poultry farm environmental samples were collected. These environmental samples consisted of litter and wastewater from various poultry farms in the Barishal region and were selected because they are common sources of *E. coli* contamination due to their potential for bacterial colonization (Figure 2).

While farm environment and poultry liver samples were initially considered as separate entities, preliminary comparison of resistance patterns between these two sample types showed no significant differences (p > 0.05). Therefore, both were grouped together under the “animal health” category for statistical analysis. This approach reflects the interconnected nature of AMR transmission in poultry production environments, where bacteria can be transmitted not only through direct infection (liver) but also via environmental contamination (e.g., litter, wastewater). By combining these samples, the study aimed to provide a comprehensive analysis of AMR risk in poultry, incorporating both direct infection sources and environmental reservoirs.

Human health samples: A total of 125 human urine samples were collected from patients clinically diagnosed with UTIs in hospital settings. These patients were diagnosed based on clinical symptoms of UTIs and confirmed by healthcare providers. The urine samples were collected using the clean-catch midstream method, which is crucial to reduce contamination from the skin and genital area. Each patient was instructed on proper sample collection techniques to ensure accuracy and minimize contamination. Approximately 4–5 mL of urine was collected from each patient in sterile tubes and transported to the laboratory immediately after collection for processing (Figure 2). Urine samples were transported immediately after collection at 4°C for processing to maintain sample quality.

### Isolation and identification of *E. coli*

Animal, human, and environmental health samples were cultured on MacConkey agar (Oxoid, Thermo Fisher Scientific, Basingstoke, UK) supplied by Biosciences Bangladesh and incubated aerobically at 37 ± 1°C for 18–24 h to allow bacterial growth. The presumptive *E. coli* colonies (three typical colonies per plate), which are typically pink lactose-fermenting colonies, were then subcultured onto Eosin Methylene Blue agar (Oxoid) for further differentiation. Colonies with a characteristic metallic green sheen were identified as *E. coli*. Confirmation was performed using standard biochemical tests, including the IMViC series (Indole, Methyl Red, Voges–Proskauer, and Citrate utilization tests), following established microbiological protocols. The standard interpretations were Indole +, MR +, VP –, Citrate –, confirming *E. coli*, following the protocol described in Edwards and Ewing’s Identification of Enterobacteriaceae. In addition to ATCC 25922, positive and negative controls were used for each biochemical test to ensure accuracy of the results (Figure 2).

### Antimicrobial susceptibility testing

Antimicrobial susceptibility testing was performed using the Kirby–Bauer disk diffusion method. The guidelines followed were Clinical and Laboratory Standards Institute (CLSI) M100, 30th edition (2020), with intermediate results classified as resistant for the purposes of this study [23–25]. Briefly, bacterial suspensions of *E. coli* were prepared to match a 0.5 McFarland turbidity standard. The suspension was spread uniformly on Mueller–Hinton agar (Oxoid) using a sterile swab.

Although a broader range of antibiotics was routinely tested in both laboratories, this study focused on three antibiotics consistently included in regional surveillance programs across both sectors: AMP (10 μg), CIP (5 μg), and AZM (15 μg). These antibiotics were selected to enable direct cross-sectoral comparison of resistance patterns.

The antibiotic disks were placed on the agar surface, and the plates were incubated at 37°C for 18–24 h. After incubation, zones of inhibition were measured. For AMP (10 μg): susceptible (S) ≥ 17 mm, resistant (R) ≤ 13 mm; for CIP (5 μg): S ≥ 26 mm, R ≤ 20 mm. For AZM (15 μg), as CLSI or European Committee on Antimicrobial Susceptibility Testing (EUCAST) does not provide established disk diffusion breakpoints for Enterobacterales, zone diameters were interpreted based on relevant published literature [26] and should be interpreted with caution. In this study, isolates with AZM zone diameters ≥ 13 mm were provisionally categorized as susceptible.

To ensure validity and accuracy, *E. coli* ATCC 25922 was used as the quality control strain for each batch of tests. The inhibition zone diameters for the control strain consistently fell within acceptable ranges specified by CLSI guidelines: AMP (10 μg): 16–22 mm; CIP (5 μg): 29–37 mm; AZM (15 μg): 20–26 mm. This confirms that testing conditions, media, and disks were performing within specifications (Figure 2).

### Statistical analysis

Resistance proportions for each antibiotic were calculated separately for human and animal health isolates, including farm environment samples categorized under animal health. As this was a retrospective study based on available surveillance samples, no prior sample size or statistical power calculation was performed. Post hoc inclusion and exclusion criteria were applied to ensure comparability between datasets. Samples from patients with incomplete clinical data or missing identifiers were excluded, and poultry samples not meeting minimum quality standards were also excluded.

Resistance data were treated as binary variables, with isolates classified as either resistant or non-resistant for each antibiotic tested. Descriptive statistics were used to summarize findings, and comparative resistance profiles were visualized using grouped bar plots.

Univariate associations between host source and resistance status were evaluated using Fisher’s exact or Chi-square test. Statistical significance was defined at p < 0.05.

To assess the impact of host source, a logistic regression model was developed to estimate the odds of resistance to AMP, CIP, and AZM while considering combined effects of infection sources and environmental exposure under animal health. Regression estimates were summarized using forest plots.

PCA was used as an exploratory multivariate approach to examine overall patterns in AMR profiles of *E. coli* isolates. PCA reduced dimensionality and identified dominant sources of variation based on susceptibility to the selected antibiotics. The first two principal components were retained for visualization. Prior to PCA, variables were standardized (z-score transformation). The number of components retained was based on eigenvalues greater than 1 and scree plot inspection. PC1 and PC2 were retained. No rotation was applied.

### Software

All statistical analyses were performed using Stata/SE version 13.0 (StataCorp, College Station, TX, USA). Data visualization and figure generation were carried out using R version 4.5.0 within the RStudio platform. The following R packages were utilized: ggplot2, forestplot, and factoextra. ArcGIS version 10.8 (Environmental Systems Research Institute, Inc., Redlands, CA, USA) was used to construct maps.

## RESULTS

### Sample distribution and isolation of *E. coli*

In this study, a total of 217 samples were analyzed, including 125 human urine samples from patients with UTIs and 92 animal-associated samples, which included liver and farm environmental specimens. *E. coli* was isolated from 68 of the 125 human health samples and 49 of the 92 animal health samples, corresponding to prevalences of 54.4% and 53.3%, respectively. Among animal-associated samples, *E. coli* was isolated from 31 of 65 poultry liver samples (47.7%) and 18 of 27 poultry farm environmental samples (66.7%). All confirmed *E. coli* isolates were subsequently included in antimicrobial susceptibility testing and statistical analyses.

### Antibiotic susceptibility patterns

Among animal health isolates, resistance was highest to AMP (47%), followed by CIP (29%) and AZM (8%). The proportion of isolates with intermediate susceptibility was 45% for AMP, 24% for CIP, and 29% for AZM. In contrast, the percentage of susceptible isolates among animal samples was 8% for AMP, 47% for CIP, and 63% for AZM.

In comparison, human health isolates exhibited markedly higher resistance levels. Resistance to AMP was highest, with 87% of isolates classified as resistant, while resistance to CIP and AZM was 40% and 56%, respectively. The proportion of isolates with intermediate susceptibility among human samples was low, recorded at 6% for AMP and 1% for AZM, whereas no intermediate isolates were observed for CIP. The percentage of susceptible isolates among human isolates was 7% for AMP, 60% for CIP, and 43% for AZM.

**Figure 3:**
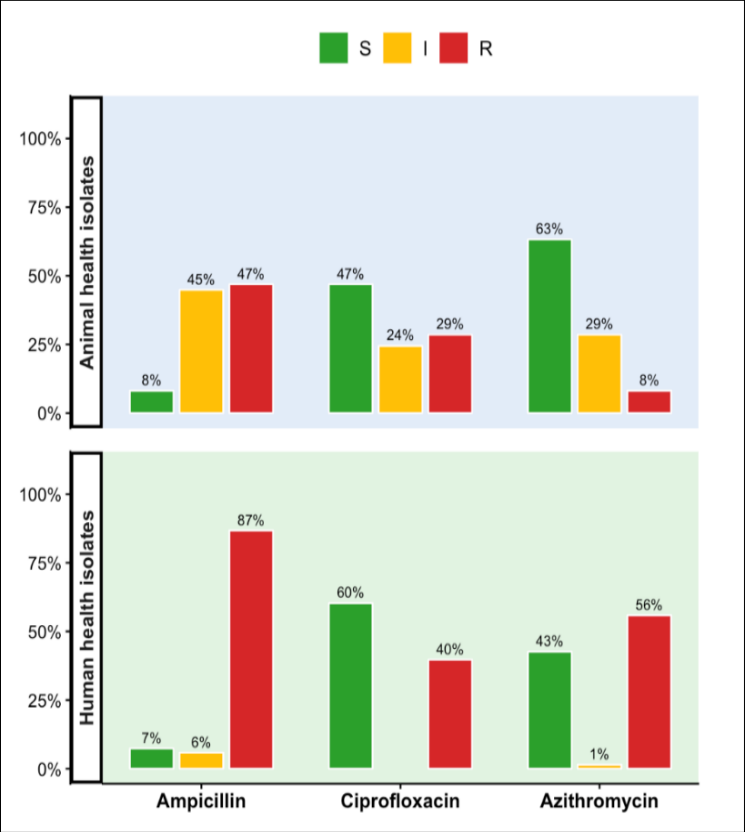
Antibiotic susceptibility patterns of *Escherichia coli* isolates in animal (n = 49) and human (n = 68) health.

For analytical purposes, isolates showing intermediate susceptibility were combined with resistant isolates and considered resistant in subsequent univariate and multivariate analyses (Figure 3).

### Univariate association

Univariate analysis confirmed significant associations between host source and resistance to specific antibiotics (Table 1). Resistance to AMP was significantly more common among human-derived *E. coli* isolates than among animal-associated isolates (86.8% vs. 46.9%; p < 0.001). A similar pattern was observed for AZM, with markedly higher resistance among human isolates compared with animal isolates (55.9% vs. 8.2%; p < 0.001). In contrast, resistance to CIP did not differ significantly between human and animal-associated isolates (39.7% vs. 28.6%; p = 0.243).

**Table 1:** Univariate association of antibiotic resistance between animal and human samples.

Variables	Animal % (n)	Human % (n)	p-value (Exact/Chi-square)
AMP	46.9 (23)	86.8 (59)	< 0.001
CIP	28.6 (14)	39.7 (27)	0.243
AZM	8.2 (4)	55.9 (38)	< 0.001

AMP = Ampicillin, CIP = Ciprofloxacin, AZM = Azithromycin.

### Multivariate logistic regression

**Figure 4:**
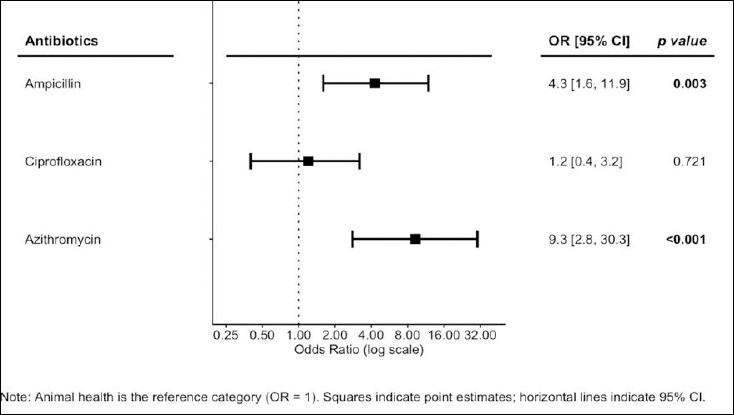
Multivariate logistic regression analysis of antibiotic resistance between animal and human health isolates.

Multivariable logistic regression analysis revealed that *E. coli* isolates from human samples had significantly higher odds of resistance to AMP and AZM compared with poultry-associated isolates, with odds ratios of 4.3 (95% CI: 1.6–11.9; p = 0.003) and 9.3 (95% CI: 2.8–30.3; p < 0.001), respectively. In contrast, no statistically significant association was observed between host source and CIP resistance in the multivariable model (p = 0.243) (Figure 4).

### PCA

**Figure 5:**
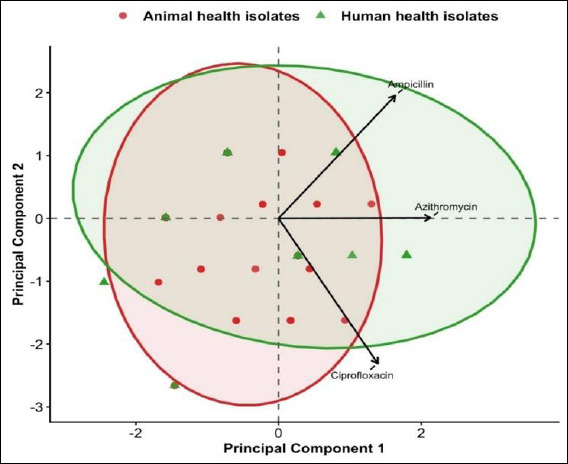
PCA biplot of AMR profiles among animal and human health isolates.

PCA was performed to explore multivariate patterns in AMR profiles and to assess similarities and differences between human and animal health isolates (Figure 5). PCA identified three principal components explaining 100% of the total variance in the dataset (Table 2). The first principal component (PC1) accounted for 48.2% of the total variance and was primarily driven by resistance to AMP and AZM, which showed the highest positive loadings on this component (0.59 and 0.70, respectively) (Table 3). The second principal component (PC2) explained an additional 33.0% of the variance and was mainly influenced by CIP resistance (loading = 0.84), with a negative contribution from AMP resistance (−0.53). Together, PC1 and PC2 explained 81.2% of the overall variance in AMR patterns. The third principal component (PC3), explaining the remaining 18.8%, showed a negative correlation with AZM resistance (−0.71) and a positive association with AMP resistance (0.59), reflecting complex interactions of these antibiotics in AMR patterns (Tables 2 and 3).

**Table 2:** Eigenvalues and variance explained by principal components.

Component	Eigenvalue	Proportion of variance explained (%)	Cumulative variance (%)
Comp1	1.44	48.2	48.2
Comp2	0.99	33.0	81.2
Comp3	0.56	18.8	100

**Table 3:** Component loadings (eigenvectors).

Variable	Component 1	Component 2	Component 3
AMP	0.59	−0.53	0.59
CIP	0.38	0.84	0.36
AZM	0.70	−0.01	−0.71

AMP = Ampicillin, CIP = Ciprofloxacin, AZM = Azithromycin.

## DISCUSSION

### Overview of cross-sectoral AMR patterns

This study provides a comprehensive cross-sectoral analysis of AMR patterns in *E. coli* isolates from both human and animal health settings in the Barishal District of southern Bangladesh, utilizing routine surveillance data. The findings highlight significant differences in resistance patterns across these sectors and emphasize the need for integrated AMR monitoring and coordinated antimicrobial stewardship efforts across both human and animal health systems, particularly in low- and middle-income countries. To our knowledge, this is among the first studies in southern Bangladesh to provide a direct phenotypic comparison of AMR in *E. coli* isolates from human UTIs, poultry diagnostic liver samples, and farm environmental sources within a shared geographic cluster (approximately 10 km radius in Barishal District). This approach demonstrates the feasibility and value of integrating existing laboratory networks for One Health surveillance in resource-limited settings, offering a practical model that can be replicated in other districts of Bangladesh and similar low- and middle-income countries.

### Human health AMR patterns

In human health isolates, resistance was notably higher, with 86.8% resistant to AMP, 39.7% resistant to CIP, and 55.9% resistant to AZM. AMP resistance in UTI pathogens is high in Bangladesh, with resistance rates consistently ranging from 70% to 90% [27–31]. This high resistance is primarily attributed to extended-spectrum beta-lactamase and other beta-lactamase genes, often located on mobile genetic elements such as plasmids [32]. These elements facilitate the spread of resistance genes among bacterial populations, contributing to cross-resistance among antibiotic classes. For instance, resistance to AMP is often linked with co-resistance to other beta-lactams, such as cephalosporins, due to the presence of beta-lactamase genes [33].

Similarly, AZM resistance is increasing, contributing to an overall multidrug resistance (MDR) rate of 77.6% [34]. The inappropriate use of AZM, both in human healthcare and veterinary settings, has exacerbated this issue. CIP resistance is also prevalent, with *E. coli* resistance exceeding 40–50% in regions such as Asia and Africa [21, 35–36]. This rising resistance is frequently associated with mutations in the quinolone resistance-determining regions of *gyrA* and *parC*, as well as plasmid-mediated *qnr* genes, which confer CIP resistance [37–39].

### Animal health AMR patterns

In animal health, 46.9% of isolates showed resistance to AMP, 28.6% to CIP, and 8.2% to AZM, with AMP resistance in poultry reaching high levels. *E. coli* isolates from broiler cloacae and layer farms exhibited 100% resistance [40–42], often accompanied by MDR. Surveys of chicken meat revealed AMP resistance in *E. coli* ranging from 82% to 100% [41]. This widespread resistance is consistent with patterns observed in settings where antibiotic use in poultry production is common [43].

AZM resistance was observed despite regulatory prohibitions on its use in veterinary medicine in Bangladesh [44]. Macrolide resistance, including AZM, was observed in 58.8% of *E. coli* isolates [45], while CIP resistance in poultry is similarly high, with studies reporting resistance levels up to 71%. These patterns highlight the potential for transmission between reservoirs through contaminated meat, eggs, direct contact, or environmental pathways such as manure-contaminated water.

### Statistical interpretation and PCA findings

These resistance patterns were further supported by multivariable logistic regression, which revealed significantly higher odds of resistance in human health isolates compared to animal health isolates. This difference may reflect distinct selective pressures operating in human and animal health settings [46, 47]. The widespread, often empirical use of AZM and other antibiotics in human healthcare has contributed significantly to the development of resistance [48, 49].

While recent studies in Bangladesh have reported uniformly high CIP resistance across human and animal sectors [21], the finding of non-significant differences between human (39.7%) and animal (28.6%) isolates (*p* = 0.243) provides an important insight into antibiotic-specific selective pressures in this setting. The absence of a significant sector difference for CIP, in contrast to the marked disparities observed for AMP and AZM, suggests that fluoroquinolone resistance may be more evenly disseminated across the human–animal–environment interface in Barishal.

This is further supported by PCA findings, where CIP resistance loaded predominantly on PC2 (loading = 0.84) and showed a negative correlation with AMP resistance (−0.53), indicating that CIP resistance follows a distinct epidemiological trajectory from beta-lactam and macrolide resistance in this setting.

PCA further identified AMP and AZM resistance as the primary drivers of variation in the dataset, explaining 81.2% of the total variance. This strong association suggests that resistance to these antibiotics, driven by mechanisms such as beta-lactamase production for AMP [50] and ribosomal methylation or efflux pumps for AZM [51], plays a central role in shaping the broader AMR landscape. The dominance of these antibiotics in explaining variance suggests overlapping resistance determinants may be circulating across human and animal health settings [52]. These findings underscore the importance of prioritizing surveillance and interventions targeting these antibiotics [46]. Interventions aimed at reducing the use of AMP and AZM could significantly lower the overall AMR burden. Furthermore, the strong link between resistance to these antibiotics and broader MDR profiles supports their use as sentinel indicators for monitoring AMR trends in both clinical and veterinary contexts [53].

### Regulatory effectiveness and challenges

Regulatory changes in Bangladesh, such as the prohibition of antibiotics in animal feed [54] and the ban on the use of AZM in veterinary medicine [55], have been implemented to reduce AMR in animal populations. However, this study was not designed to assess the impact of these regulations, and no pre-ban baseline data were available for comparison. The continued detection of AZM resistance (8.2% in animal isolates) suggests either residual resistance from prior use, co-selection with other antibiotics, or potential gaps in enforcement at the local level in Barishal. The prevalence of resistance genes, despite these regulatory efforts, highlights the need for stricter enforcement, robust monitoring systems, and future studies specifically designed to evaluate the effectiveness of such interventions over time.

### Global context and One Health perspective

The findings align with global AMR trends reported in other low- and middle-income countries. In Bangladesh, the high levels of AMR highlight the urgency of coordinated interventions across human, animal, and environmental sectors. Adopting a One Health approach is critical, linking resistance patterns to shared ecological and environmental factors. To strengthen policy relevance, these results are explicitly connected to Bangladesh’s NAP on AMR, emphasizing integrated strategies across sectors. The importance of WASH (Water, Sanitation, and Hygiene) practices in poultry farms is also highlighted to reduce environmental contamination and limit cross-sectoral AMR transmission. This underscores the need for practical, cross-sectoral One Health interventions combining surveillance, regulatory enforcement, biosecurity, and environmental management to mitigate AMR in Bangladesh and similar settings [52].

### Study limitations

This study has several limitations. The analysis was based on cross-sectional data derived from routine regional government surveillance and followed a retrospective sampling approach. As samples were obtained through routine surveillance, there is potential for selection bias, as sampling may have targeted specific farms or clinical settings rather than following a randomized design. In addition, human and animal samples were not temporally matched, which may affect the interpretation of cross-sectoral resistance patterns.

The study focused on only three antibiotics (AMP, CIP, and AZM) that were consistently tested across sectors to allow direct comparison. Farm environment and poultry liver samples were grouped under “animal health” due to their common origin, reflecting interconnected AMR transmission; however, this may have masked differences between sample types. Due to the retrospective design, detailed epidemiological and clinical information was not consistently available, limiting generalizability. Direct antimicrobial use data were also lacking, and the cross-sectional design prevents establishing directionality of transmission.

Environmental sampling was limited to farm litter and wastewater; broader environmental sources were not included. The absence of genotyping (e.g., WGS [Whole Genome Sequence] and MLST [Multilocus Sequence Typing] means clonal relatedness and gene transfer events cannot be confirmed. Additionally, phenotypic identification alone may have limitations, as rare atypical strains of *E. coli* may not exhibit typical biochemical profiles. No formal sample size calculation was performed, and the study may have been underpowered to detect smaller differences, particularly for CIP resistance (p = 0.243).

### Future research directions

These findings support the urgent need to expand surveillance to include molecular typing approaches such as WGS and MLST to confirm clonal relatedness and identify transmission pathways between reservoirs. Future research should focus on characterizing mobile genetic elements and resistance genes (e.g., *bla*_TEM_, *bla*_CTX-M_, *qnr*, *mef*) to elucidate the genetic mechanisms underlying sector-specific patterns.

Longitudinal studies with temporally matched sampling across sectors and inclusion of a broader range of antibiotic classes are needed to capture the full spectrum of AMR dynamics and inform targeted interventions aligned with the NAP. Further research should also address environmental drivers of AMR, including contamination of wastewater and soil.

Additionally, evaluating the effectiveness of interventions targeting the overuse of AZM and other antibiotics in both human and animal health settings will be essential. Given the high levels of AMP resistance observed, future investigations should include phenotypic screening and molecular detection of beta-lactamase genes (e.g., *bla*_TEM_, *bla*_CTX-M_, *bla*_CMY_) to better understand resistance mechanisms and their potential for transmission.

## CONCLUSION

This study provides clear evidence of sector-specific differences in AMR patterns of *E. coli* in southern Bangladesh. Human health isolates exhibited substantially higher resistance to AMP (86.8%) and AZM (55.9%) than animal-associated isolates (46.9% and 8.2%, respectively), whereas CIP resistance showed no statistically significant difference between sectors (p = 0.243). Multivariable analysis further confirmed significantly higher odds of resistance to AMP and AZM in human isolates, and PCA demonstrated that these two antibiotics were the primary drivers of variability, collectively explaining 81.2% of the observed variance. These findings highlight the dominant role of beta-lactam and macrolide resistance in shaping the AMR landscape in this setting.

From a practical perspective, the observed resistance patterns emphasize the urgent need to strengthen antimicrobial stewardship, particularly in human healthcare, where selective pressure appears to be highest. The high resistance to AMP and AZM suggests that these antibiotics should be prioritized for surveillance and rational use interventions. In addition, the comparable levels of CIP resistance across sectors indicate widespread dissemination across the human–animal–environment interface, underscoring the need for integrated One Health surveillance systems. Strengthening regulatory enforcement, improving prescribing practices, enhancing farm biosecurity, and promoting WASH interventions are critical to limiting cross-sectoral transmission of resistant bacteria.

A key strength of this study lies in its cross-sectoral design, integrating human clinical samples, animal diagnostic samples, and farm environmental sources within a shared geographic setting. This approach provides a realistic representation of AMR dynamics in a resource-limited context and demonstrates the feasibility of utilizing existing surveillance infrastructure for One Health monitoring. The use of multiple analytical approaches, including logistic regression and PCA, further strengthens the reliability of the findings by providing both statistical and multivariate insights into resistance patterns.

In conclusion, this study highlights the urgent need for coordinated, evidence-based interventions targeting key resistance drivers such as AMP and AZM. The findings support implementing integrated One Health strategies to improve AMR surveillance, optimize antibiotic use, and reduce transmission across sectors. Addressing AMR in settings such as Bangladesh requires sustained collaboration between human, animal, and environmental health systems to safeguard the effectiveness of existing antimicrobials and protect public health.

## DATA AVAILABILITY

All the generated data are included in the manuscript.

## AUTHORS’ CONTRIBUTIONS

IK and MMH: Conceived, designed, and coordinated the study, and developed data collection tools. RAD, IK, MNA, AKMAK, KNK, MJH, and NP: Oversaw field sampling, data collection, laboratory work, and data entry. RAD, IK, and MMH: Conducted project management, statistical analysis, interpretation, and manuscript writing. MSH: Data visualization. IK, RAD, MSH, MNA, AKMAK, KNK, NP, and MMH: Performed a critical review of the manuscript and ensured compliance with ethical protocols. All authors read and approved the final version of the manuscript. RAD and IK contributed equally as first authors.
